# Dominant oceanic bacteria secure phosphate using a large extracellular buffer

**DOI:** 10.1038/ncomms8878

**Published:** 2015-07-22

**Authors:** Mikhail V. Zubkov, Adrian P. Martin, Manuela Hartmann, Carolina Grob, David J. Scanlan

**Affiliations:** 1 National Oceanography Centre, Ocean Biogeochemistry & Ecosystems Research Group, European Way, Southampton SO14 3ZH, UK; 2 School of Life Sciences, University of Warwick, Gibbet Hill Road, Coventry CV4 7AL, UK

## Abstract

The ubiquitous SAR11 and *Prochlorococcus* bacteria manage to maintain a sufficient supply of phosphate in phosphate-poor surface waters of the North Atlantic subtropical gyre. Furthermore, it seems that their phosphate uptake may counter-intuitively be lower in more productive tropical waters, as if their cellular demand for phosphate decreases there. By flow sorting ^33^P-phosphate-pulsed ^32^P-phosphate-chased cells, we demonstrate that both *Prochlorococcus* and SAR11 cells exploit an extracellular buffer of labile phosphate up to 5–40 times larger than the amount of phosphate required to replicate their chromosomes. Mathematical modelling is shown to support this conclusion. The fuller the buffer the slower the cellular uptake of phosphate, to the point that in phosphate-replete tropical waters, cells can saturate their buffer and their phosphate uptake becomes marginal. Hence, buffer stocking is a generic, growth-securing adaptation for SAR11 and *Prochlorococcus* bacteria, which lack internal reserves to reduce their dependency on bioavailable ambient phosphate.

## Abstract (web summary)

Oceanic SAR11 Alphaproteobacteria and *Prochlorococcus* cyanobacteria are abundant in phosphate-poor regions, despite it being vital for growth. Here, Zubkov *et al.* show these bacterioplankton exploit an extracellular buffer of labile phosphate to reduce their dependency on bioavailable ambient phosphate.

## Graphical abstract

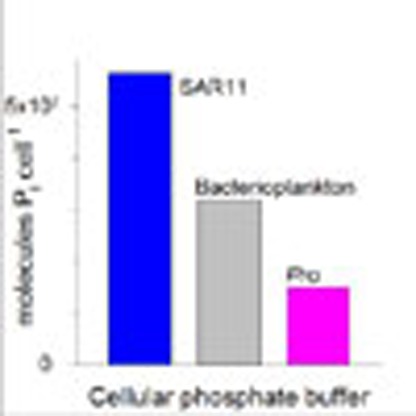

Phosphorus, a vital element for cellular energetics and growth as well as the ultimate controller of oceanic productivity^1^, is acquired by oceanic bacterioplankton primarily as phosphate^2^,^3^. Strongly stratified surface seawater can be depleted in phosphate down to concentrations of a few nanomoles per litre^4^,^5^,^6^. Despite this, the dominant bacterioplankton^7^ of these low-phosphate regions, the SAR11 alphaproteobacteria^8^ and *Prochlorococcus* cyanobacteria^9^, are almost as abundant and metabolically active as anywhere else in the ocean^10^,^11^. Somehow these bacteria maintain a sufficient supply of phosphate in apparently phosphate-poor waters.

We show that the rates of phosphate uptake by SAR11 and *Prochlorococcus* cells vary by an order of magnitude from the temperate to the equatorial North Atlantic, whilst their amino-acid and ATP uptake rates, as well as CO_2_ fixation rates by *Prochlorococcus* cells, remain constant. Their phosphate uptake is counter-intuitively lower in more productive tropical waters, as if cellular demand for phosphate decreases there. This contradiction is resolved using a novel ^33^P-phosphate pulse ^32^P-phosphate chase experimental approach. By flow sorting dual-labelled cells, we demonstrate that both *Prochlorococcus* and SAR11 cells possess an extracellular buffer of labile phosphate.

## Results

### Identification of bacterioplankton dominant groups

An experimental field study was carried out on the two Atlantic Meridional Transect (AMT) research cruises with similar tracks across the North Atlantic subtropical gyre in October–November 2010 and 2012 (Supplementary Fig. 1).

Microbial cells, flow sorted from the two main groups of bacterioplankton during the 2010 cruise, were taxonomically identified using fluorescence *in situ* hybridization^7^. High-nucleic acid-containing bacteria, based on DNA staining with SYBR Green I that had virtually undetectable chlorophyll autofluorescence^12^, were phylogenetically affiliated with *Prochlorococcus*^7^, in agreement with our previously reported results^2^. An average of 88±4% (*n*=10) of flow-sorted, DNA stained with 4,6-diamidino-2-phenylindole (DAPI) cells conferred signals with the *Prochlorococcus*-specific probe PRO405 (ref. 13). The majority of the flow-sorted cells with low nucleic acid content affiliated to the SAR11 clade^7^; 93±6% (*n*=9) of DAPI-stained cells were positively identified using a set of probes that target different regions of the SAR11 rRNA improving our earlier result^2^ and allowing us to refer to the low-nucleic acid-containing bacteria as SAR11 alphaproteobacteria. On the basis of the molecular identification of the two bacterioplankton populations, SAR11, followed by *Prochlorococcus*, numerically dominate surface waters of the North Atlantic subtropical gyre (Supplementary Fig. 2).

### Microbial uptake of bioavailable nutrients

**Figure 1:**
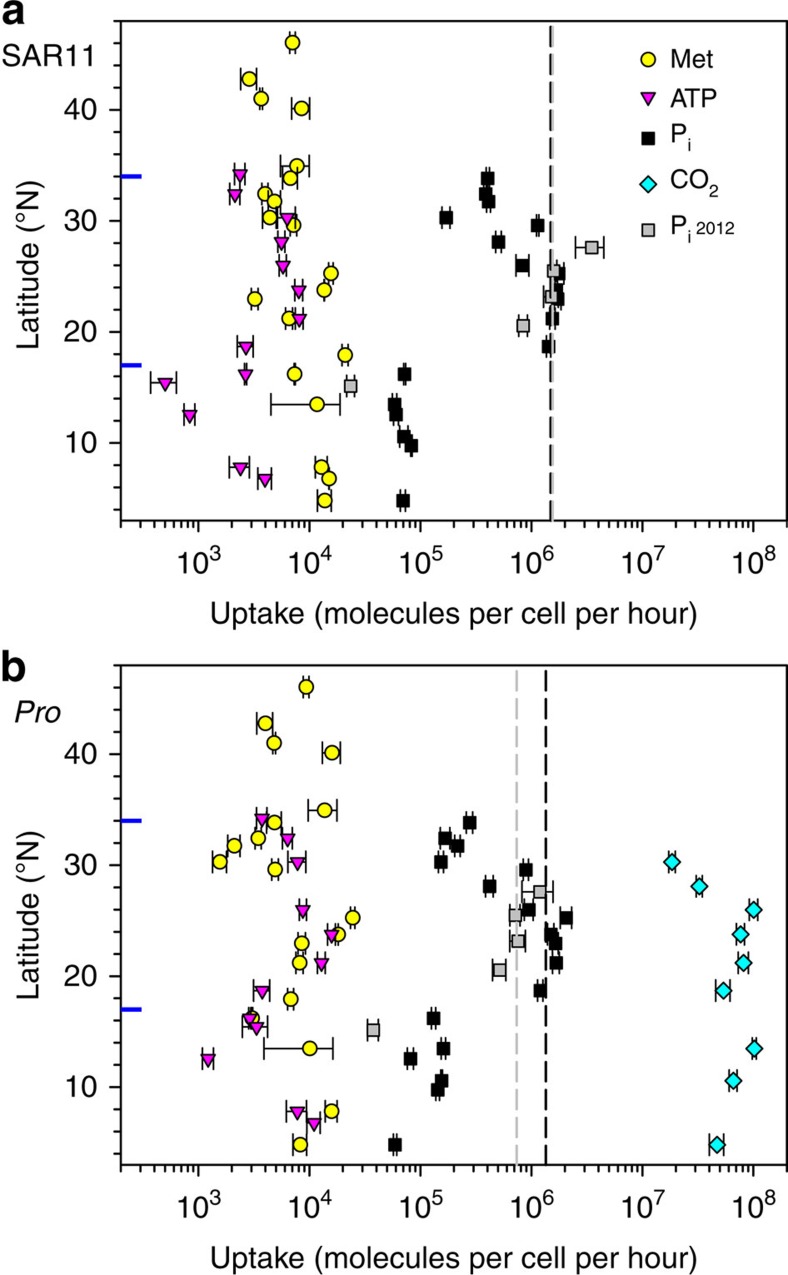
Latitudinal distribution of cellular nutrient uptake and CO_2_ fixation rates of the two dominant bacterioplankton groups. Latitudinal distribution of nutrient uptake rates of SAR11 (**a**) and of nutrient uptake and CO_2_ fixation rates of *Prochlorococcus* (*Pro*) (**b**) cells in the North Atlantic subtropical gyre on the 2010 cruise. The nutrients were methionine (Met), ATP and inorganic phosphate (P_i_). Only cellular uptake rates of inorganic phosphate (P_i_ 2012) were measured on the 2012 cruise. Black and grey dashed lines indicate median rates of P_i_ uptake in the phosphate-depleted centre of the gyre during the 2010 and 2012 cruises, respectively. Blue horizontal tick lines indicate boundaries of the central gyre. Error bars indicate propagated standard errors of combined measurements of microbial nutrient uptake rates, cell abundances and radiotracer concentrations in flow-sorted cells.

We used the modified Wright and Hobbie bioassay technique^2^,^14^ to estimate concentrations and microbial uptake rates of phosphate, amino acid—methionine (indication of microbial growth)—and ATP (indication of microbial organic phosphate acquisition; Supplementary Figs 3–5, see Methods for details). We flow sorted radiotracer-labelled cells to quantify *Prochlorococcus* and SAR11 group-specific uptake rates. First, we compared cellular uptake rates of phosphate with cellular uptake rates of other molecules to ascertain the extent of misbalance of molecule acquisition (Fig. 1), that is, do the cellular uptake rates of other molecules like amino acids or ATP or CO_2_ fixation (indication of phytoplankton growth) also decrease in more productive equatorial waters? Second, we carried out experiments using a ^33^P-phosphate pulse followed by a ^32^P-phosphate chase to determine the potential role of extracellular buffer in phosphate acquisition by *Prochlorococcus* and SAR11 (Supplementary Fig. 1, large and medium yellow squares).

According to the isotope dilution bioassay and cell flow sorting results of the 2010 cruise, uptake rates of methionine and ATP by SAR11 cells are relatively constant, varying within an order of magnitude. The uptake rates of phosphate by SAR11 cells contrasted with their uptake rates of both ATP and methionine (Fig. 1a), in that there was a 15-fold drop in cellular phosphate uptake at the boundary between the gyre centre and more populous, productive and nutrient-rich tropical surface waters (Supplementary Figs 1–2,4–5). The drop in phosphate uptake of SAR11 cells was reproducible on the two cruises and coincided with an increase in ambient phosphate uptake time (turnover) to >10 h (Supplementary Fig. 4c).

Constancy of their methionine uptake rate indicates that SAR11 cells synthesize their proteins at comparable rates across the North Atlantic. However, they seem to need less phosphate for their growth in more productive waters. On the basis of the size of the representative *Pelagibacter ubique* genome^15^, a SAR11 cell requires 2.6 × 10^6^ molecules of phosphate for its chromosome replication. According to our results, a SAR11 cell could acquire that amount of phosphate in ∼2.5 h in the gyre centre, whilst it would take one nearly 38 h to acquire the same amount of phosphate in tropical waters at the rates measured. If we assume that phosphate quota in DNA is about one-fourth of the amount of phosphate required for producing a daughter cell, then hypothetically SAR11 cells could divide twice per day in the gyre and every 6 days in the tropical waters. Hence, based on phosphate uptake data, SAR11 cells should grow slower in tropical waters than in the gyre centre. This is in contradiction to the results of their methionine uptake, according to which they should grow at a similar rate.

The second dominant bacterioplankton cells, *Prochlorococcus* cyanobacteria, show similar reduction in the uptake of phosphate relative to their uptake rates of ATP and methionine as well as relative to their CO_2_ fixation rates in tropical waters compared with the gyre centre (Fig. 1b). Similar to SAR11 cells, the uptake rate of phosphate by *Prochlorococcus* cells decreased abruptly by eight times. On the basis of the size of the *Prochlorococcus marinus* genome^16^, a *Prochlorococcus* cell could acquire the 3.3 × 10^6^ molecules of phosphate required for chromosome replication in ∼3.3 h in the gyre centre, whilst it would take a *Prochlorococcus* cell 27 h to acquire the same amount of phosphate in tropical waters. Making a similar assumption that there is about a quarter of cellular phosphate in DNA, then hypothetically *Prochlorococcus* cells could divide twice a day in the gyre and every 4—5 days in tropical waters, which is completely opposite to what we observed in the South Atlantic gyre^17^.

For *Prochlorococcus*, the corresponding molar ratio between hourly CO_2_ fixation and phosphate uptake would be 65 and 530 in the gyre centre and tropical waters, respectively. Taking into account that these cyanobacteria can fix CO_2_ only during the hours of daylight while they can take up phosphate 24 h a day, the first ratio is closer to the Redfield molar elemental ratio of 106 (refs 18, 19). The second ratio of 530 (or double this allowing for the difference in periods of acquisition) is an order of magnitude different from the Redfield ratio, suggesting that normal growth of *Prochlorococcus* cells in more productive tropical waters could be impeded by a deficit in phosphorus. The latter conclusion would not explain the observed higher concentrations of *Prochlorococcus* cells there (Supplementary Fig. 2). The consistent drop in phosphate uptake rate by SAR11 and *Prochlorococcus* cells in tropical waters is unlikely to be due to a decrease in their metabolic activity because the rates of uptake of the other molecules studied remained unchanged whilst the concentrations of cells increased. Furthermore, the concentration of bioavailable phosphate in surface tropical waters remained similar or increased but by no more than three times (Supplementary Fig. 4). Therefore, the drop in phosphate uptake rate by these microorganisms is not related to cell growth.

### Assessing microbial extracellular phosphate buffer

A plausible explanation for the reduction in uptake of phosphate in tropical waters could be the existence of large extracellular phosphate buffers, because SAR11 and *Prochlorococcus* do not store phosphate in cells^20^,^21^. To assess how significant such a microbial extracellular phosphate buffer is, we first carried out experiments using a ^33^P-phosphate pulse, chased with unlabelled phosphate (Supplementary Fig. 6a). The results of these experiments consistently show a slowing down of ^33^P-phosphate uptake after addition of the chase at 1,000 times higher concentration compared with the pulse (Supplementary Fig. 6b). Nevertheless, the uptake of ^33^P-phosphate pulse continued steadily during ≥8 h after the chase was added. The possibility of the effect being due to the cells switching to a lower-affinity transport system during the chase is unlikely because such transporters have not been found in *Prochlorococcus* and SAR11 genomes. Furthermore, the amount of acquired phosphate (accounting for chase dilution of the ^33^P tracer) during the chase would be approximately equivalent to cellular biomass, which is unrealistic. The existence of a large extracellular phosphate buffer associated with bacterioplankton cells yet distinct from bioavailable phosphate dissolved in seawater explains the continued uptake of ^33^P-phosphate from this buffer of phosphate tracer accumulated during the pulse. For comparison, the cellular pools of amino acids are exhausted within the first hour of chase^22^. However, in the case of phosphate, there were no signs of pulse stabilization (Supplementary Fig. 6b).

**Figure 2:**
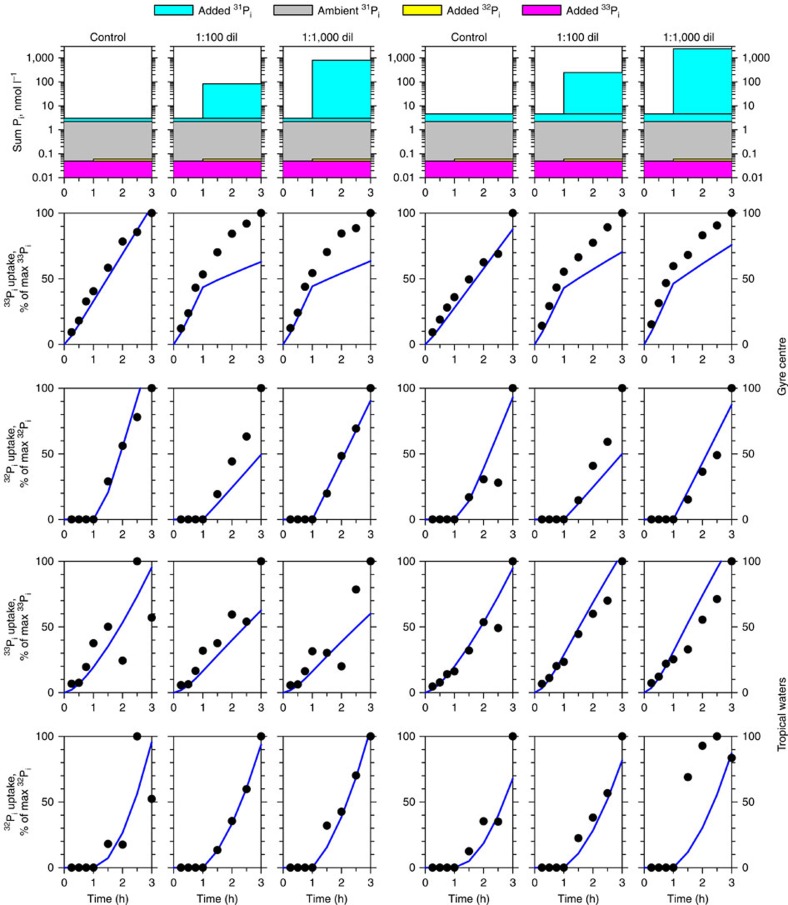
Comparison of observations and model output of inorganic phosphate uptake during ^33^P-phosphate pulse and ^32^P-phosphate chase experiments. Observations (black circles) and model output (blue lines) of traced inorganic phosphate (^33^P_i_ and ^32^P_i_) in cells during six pulse–chase experiments (top row of plots) at two contrasting sites: least productive waters of the gyre centre and more productive tropical waters. Seawater samples with bioassayed ambient ^31^P_i_ were pulsed with 0.8 or 2.4 nmol l^−1 31^P_i_ labelled with 0.05 nmol l^−1 33^P_i_ and chased after 1 h incubation with 80 or 800 nmol l^−1^ and 240 or 2,400 nmol l^−1^ of ^31^P_i_ labelled with 0.01 nmol l^−1 32^P_i_. Note that observations and model output values are normalized to the maximum uptake measure (counts per minute per ml) in a time course in each experiment to lie between 0 and 100%. dil, dilution.

To find out how cells maintain their phosphate buffers, we carried out more complex experiments in which the ^33^P-phosphate pulse was chased with higher concentrations of phosphate, but this time the chase was labelled with a ^32^P-phosphate tracer (Fig. 2, top row). An example of our approach is as follows: a seawater sample was pulsed with 0.8 nmol phosphate per l labelled with ^33^P-phosphate. After incubation at *in situ* temperature in the dark for 1 h, a chase labelled with ^32^P-phosphate was added at a concentration of 80 or 800 nmol phosphate per l and the sample was incubated for a further 2 h. In control experiments, a sample pulsed with 0.8 nmol phosphate per l labelled with ^33^P-phosphate was chased with ^32^P-phosphate tracer addition (<0.01 nmol phosphate per l). Microbial uptake of ^33^P and ^32^P tracers was followed during the pulse and chase phases of the incubation.

In agreement with the isotope dilution bioassays (Supplementary Fig. 3) similar uptake rates of the two tracers in the control chase incubations (Supplementary Fig. 7) confirmed that bacterioplankton cells did not differentiate between ^33^P- and ^32^P-labelled phosphate. If there was no labile buffer of phosphate associated with cells, then one would expect that uptake of the two phosphate tracers during the chase phase would be similar, with rate measurements close to the unity line (Supplementary Fig. 7). However, the uptake rates of the two tracers were markedly different, because the uptake of the ^33^P-phosphate pulse was not halted by the chase (Supplementary Fig. 7). These consistent results confirmed the presence of an extensive buffer of labile phosphate associated with bacterioplankton cells.

**Figure 3:**
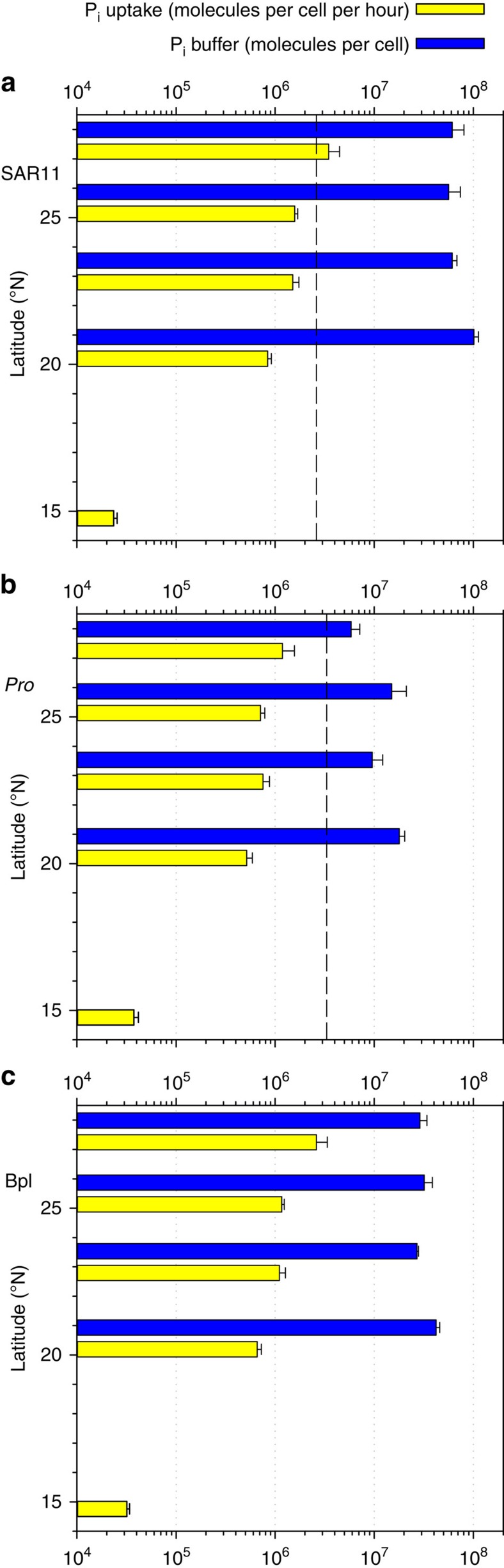
Cellular phosphate uptake rates and estimates of extracellular phosphate buffer. Comparison of measurements of uptake rates of inorganic phosphate (P_i_) with estimates of cellular phosphate buffer of SAR11 (**a**), *Prochlorococcus* (*Pro*) (**b**) and average of total bacterioplankton (Bpl) (**c**). Dashed black lines indicate the estimated amount of phosphate in SAR11 and *Prochlorococcus* chromosomes. Owing to low uptake of ^33^P_i_ tracer and background uptake of ^32^P_i_ tracer at 15° N, the phosphate buffer could not be estimated. Error bars indicate propagated errors of bioassay regression slopes, s.d. of different flow cytometric sorts and buffer estimates. To assist plot reading vertical dotted grid lines indicate main *x* axis ticks.

To further examine the hypothesis that phosphate, held in a buffer, is used by bacterioplankton cells a simple numerical model was developed to simulate uptake of phosphate by a population of cells (details in Methods section). Each cell takes up phosphate into its buffer at a rate controlled by the degree to which the buffer is full; uptake decreases as it fills and stops when the buffer is full. However, phosphate is transported from the buffer into the cell for macromolecule synthesis and energetics at constant rates reflecting cellular metabolism. Figure 2 shows that this simple model can nevertheless capture the main features of experiments at two contrasting sites (Supplementary Fig. 1, large yellow squares) simultaneously. The only model parameters allowed to vary between the two sites were the ambient phosphate concentration and the degree to which the buffer is full at the start of the experiments. Observations are used to set the initial number of cells (Supplementary Fig. 2a). Even though the predicted uptake is lower than observations, the model captures the persistently increasing ^33^P uptake when ^32^P-labelled chases are applied at 1 h. When the model did not account for a buffer, no solution was found where the ^33^P uptake did not decrease on addition of the ^32^P chase. Although care must be exercised interpreting the details of such a simple illustrative model, the parameter values needed to give an acceptable match to observations simultaneously provide an estimate for the maximum size of the buffer of a mean bacterioplankton cell as 2 × 10^7^ molecules. This estimate is not far from the experimentally determined buffer of a mean bacterioplankton cell (3.2 × 10^7^ molecules), being 3.5 times smaller compared with the buffer of SAR11 cells (7 × 10^7^ molecules) and being 1.7 times higher compared with the buffer of *Prochlorococcus* cells (1.2 × 10^7^ molecules) in the gyre centre (Fig. 3).

To assess whether the size of the buffer differs between bacterioplankton groups, total bacterioplankton, SAR11 and *Prochlorococcus* cells were flow sorted from samples fixed at different time points of the ^33^P-pulse ^32^P-chase experiments. Cells were sorted from samples fixed at the end of the pulse and at the end of the chase phases (Fig. 2). Uptake of ^33^P and ^32^P tracers was compared between flow-sorted cells of SAR11, *Prochlorococcus* and average bacterioplankton. The size of the buffer was estimated based on the uptake results of both tracers, the key parameter being the ratio of ^33^P to ^32^P uptake during the chase phase (see Methods for details).

Uptake of ^32^P-phosphate by sorted cells in chase experiments could be determined only in experiments with the lowest dilution (1:100) because of detection limitations with one exception at the station in tropical waters (Supplementary Fig. 1, bottom large square), where uptake of phosphate was particularly low (Fig. 3). Compared with the ^32^P tracer, the rate of ^33^P tracer uptake during the chase phase of both dilutions was measurable in sorted cells of all the groups at all stations and at all dilutions. This fact strongly indicates the presence of a buffer of phosphate associated with all the cells. The estimated size of the buffer exceeded the hourly amount of phosphate uptake by SAR11 and *Prochlorococcus* by on average of 53 and 18 times, respectively (Fig. 3a,b). Because cumulatively the two groups accounted for the majority (∼70%) of bacterioplankton (Supplementary Fig. 2), it was reassuring that the buffer for mean bacterioplankton cells exceeded the hourly amount of phosphate uptake by the intermediate average of 32 times (Fig. 3c). Indirectly, it implies that bacterioplankton cells in other taxa could also have sizable phosphate buffers. In other words, cellular phosphate buffers could be common among bacterioplankton.

For SAR11 cells the estimated phosphate buffer was 27 times the amount of phosphate required for genomic DNA replication (Fig. 3a). The phosphate buffer of *Prochlorococcus* cells was smaller, on average 3.6 times the amount of phosphate in genomic DNA (Fig. 3b). The phosphate buffer of SAR11 cells was consistently bigger than the buffer of *Prochlorococcus* cells (paired *t*-test, *P*<0.05). This implies that SAR11 cells may have a higher phosphate buffer capacity. However, we would exercise caution in generalizing the ecological significance of the difference considering the limited number of independent measurements. Furthermore, irrespective of the differences in buffer sizes, the phosphate uptake rates of SAR11 and *Prochlorococcus* cells were not different (paired *t*-test), suggesting similar phosphate acquisition efficiency of the two bacteria with different buffer capacity.

The existence of the phosphate buffer can explain the lower rates of phosphate uptake by bacterioplankton in more productive, phosphate-replete tropical surface waters (Fig. 1). The degree of saturation of the cellular phosphate buffer increases from the gyre centre to equator (Fig. 3), in parallel with the increase in concentration of bioavailable phosphate and the increase in phosphate turnover time towards the equator (Supplementary Fig. 4a,c). The above suggests that bacterioplankton do not experience much difficulty in filling their extracellular buffers in phosphate-depleted waters and consequently control the concentration of bioavailable phosphate in these strongly stratified surface waters. At 20.6° N the sum of the phosphate buffers of bacterioplankton cells was 14 times higher than the 2.3-nmol l^−1^ of bioavailable ambient phosphate. At the same time, bacterioplankton continued to take up bioavailable phosphate at a rate of 0.5 nmol l^−1^ h^−1^. A few degrees closer to the equator the bacterioplankton phosphate buffer approaches saturation, the demand for buffering phosphate is reduced and the rate of uptake of bioavailable phosphate by both SAR11 and *Prochlorococcus* cells drops by an order of magnitude, whilst their uptake rates of other molecules (methionine, ATP and CO_2_) remain high (Fig. 1).

## Discussion

Buffering should be taken into account when quantifying cellular phosphate uptake. Because labile phosphate could dissociate from fixed cells^23^, a phosphate chase is commonly used as an alternative to fixation to stabilize the amount of phosphate radiotracer in living cells^24^,^25^. However, the presented experimental evidence (Fig. 2, Supplementary Figs 6–7) suggests that such a pulse–chase approach should be used with caution. It could lead to considerable overestimation of phosphate uptake rates, because chasing with unlabelled phosphate does not stop uptake of the pulsed phosphate radiotracer.

The idea that bacteria could store phosphate as energy and growth reserves is not new—storage of phosphate inside prokaryotic as well as eukaryotic cells in the form of polyphosphate is well documented. However, neither SAR11 nor *Prochlorococcus* cells store polyphosphates^20^,^21^. Presumably they either do not have enough space inside their small cells or it is energetically untenable for them to accumulate those granules. Instead, *Prochlorococcus* and SAR11 cells seem to keep a phosphate reserve outside their cells as a buffer. The association of the phosphate buffer with bacterial cells is reversible. However, it is not a result of uncontrolled adsorption to the cell surface, because bacterial uptake of phosphate tracers is linear over a period of 1–1.5 h (Supplementary Figs 3a–b,6b), while adsorption saturates within minutes. The extracellular location of the buffer is indicated by the ease of its removal from cells. Similar to how washing *Trichodesmium* and other phytoplankton cells with oxalate removes up to 90% of adsorbed phosphate^19^, washing bacterioplankton with tri-potassium-citrate removes on average 85 and 70% of phosphate tracers from bacterioplankton cells that had been incubated with more than 20 and 0.8 nmol l^−1^ of added phosphate, respectively, before the cells were fixed (Supplementary Fig. 8a). However, when <0.05 nmol l^−1^ of ^33^P-phosphate was used for labelling cells, citrate could remove only 10% of tracer from labelled, fixed cells (Supplementary Fig. 8b). It seems that when additional amounts of phosphate as small as 0.8 nmol l^−1^ become available, bacterial cells fill their buffers presumably to secure a sufficient amount of phosphate to complete the division cycle. In contrast to nonspecific phosphate sorption to eukaryotic algal surfaces^19^ bacteria are able to readily access their buffered phosphate without compromising cell viability whilst the divided cells rapidly replenish their now-depleted phosphate buffer. Where concentrations of bioavailable phosphate are higher than a few nanomoles, for example, in the North–East Atlantic, central South Atlantic and Arabian Sea, the bacterioplankton phosphate buffer is generally full and therefore microbial phosphate uptake is close to detection limit and does not have a typical linear time course. Perhaps, the phosphate buffer is required for normal metabolism of oceanic bacterioplankton. The constantly low phosphate concentrations in the strongly stratified surface waters of the North Atlantic subtropical gyre allow the buffer and its role in microbial uptake to be delineated. The existence of the extracellular buffer also suggests that bioavailability of phosphate is most likely patchy at a micron scale of bacterioplankton environment.

Thus, the bacterioplankton that dominate the phosphate-depleted regions of the ocean exploit a large extracellular buffer to meet their phosphate requirements. Both SAR11 and *Prochlorococcus* accumulate phosphate rather than balance its uptake with the uptake of other molecules. Such a phosphate buffer could be a common feature for the majority of microbial cells. Buffering of vital nutrients explains how bacteria without internal reserves can still secure their growth.

## Methods

### Sampling and cell enumeration

The study was carried out during two transatlantic cruises, AMT-20 and AMT-22, on board the Royal Research Ships James Cook and James Clark Ross in October 2010 and 2012, respectively. Surface seawater samples were collected before dawn from a depth of 20 m, a representative depth of the surface mixed layer unaffected by the ship's movement and contamination, with a sampling rosette of 20-l Niskin bottles mounted on a conductivity–temperature–depth profiler at stations identified in Supplementary Fig. 1. Samples were gently transferred into a 10-l polyethylene carboy. All plasticware and glassware were soaked in 10% HCl and extensively rinsed with sampled sea water. Microbial cell abundances (Supplementary Fig. 2) were determined by flow cytometry (FACSort and FACSCalibur instruments, Becton Dickinson, Oxford, UK) in samples, fixed with 1% (w/v) paraformaldehyde (PFA) final concentration and stained with SYBR Green I DNA dye^26^. All experiments were set up within 20 min after sample collection.

### CO_2_ fixation by *Prochlorococcus* cells

To assess CO_2_ fixation by the smallest prokaryotic phototrophs, *Prochlorococcus* cells, trace metal-free NaH^14^CO_3_ tracer (34.66 mmol l^−1^ NaH^14^CO_3_, DHI, Denmark) was added to a 60-ml sample in a Pyrex glass bottle (250 kBq ml^−1^ final radioactivity). The sample was then incubated at ambient temperatures controlled by a refrigerated water bath (Grant Instruments, UK) in a 6-l acrylic glass water tank illuminated by a warm white light-emitting diode array (Photon Systems Instruments, Czech Republic) adjusted to a constant output of 500 μmol photons per m^2^ per s, mimicking the average *in situ* light conditions at 20 m depth in subtropical waters^7^,^12^.

### Bioassaying of bioavailable phosphate, ATP and methionine

The concentrations of bioavailable phosphate, methionine, ATP and microbial uptake rates were estimated at 20, 13 and 17 stations, respectively, on the 2010 cruise (Supplementary Figs 4–5) using an isotope dilution, concentration series bioassay^2^,^7^. L-[35S] methionine (specific activity >1,000 Ci mmol 1^−1^, Hartmann Analytic GmbH, Braunschweig, Germany) was added at a concentration of 0.05 nM and diluted with unlabelled L-methionine (Sigma Aldrich, Dorset, UK) using a dilution series spanning the range 0.05–1.0 nM. [α ^33^P]-ATP (specific activity >3,000 Ci mmol 1^−1^, Hartmann Analytic) was added at a concentration of 0.05 or 0.1 nM and diluted with non-labelled ATP–disodium salt hydrate (Sigma Aldrich) using a dilution series in the range 0.1–2.0 nM. ^33^P-phosphate tracer (specific activity 100 TBq mM^−1^, Hartmann Analytic) was added to samples at <0.05 nmol l^−1^ final concentration and diluted with unlabelled orthophosphoric acid using a dilution series spanning the range of 0.4–4.0 nM. On the 2012 cruise only the bioavailable concentration and microbial uptake of phosphate were estimated at 11 stations (Supplementary Fig. 2). Multiple replicated 1.6-ml samples were incubated in crystal clear screw cap microtubes (Starlab, Milton Keynes, UK) at *in situ* temperature in the dark and fixed with 1% (w/v) PFA.

### Microbial ^33^P-phosphate pulse, ^32^P-phosphate chase labelling

At five stations of the 2012 cruise (Supplementary Fig. 1) dual phosphate tracer pulse–chase experiments were carried out (Fig. 2). Briefly, ^33^P-phosphate tracer was added to samples at <0.05 nmol l^−1^ final concentration simultaneously with 0.8 nmol l^−1^ of unlabelled ^31^P-phosphate. After 1 h incubation in the dark at *in situ* temperature, a set of samples was fixed with 1% (w/v) PFA to determine ^33^P uptake in total samples and by flow-sorted bacterioplankton cells at the end of the pulse. To the remaining samples, <0.01 nmol l^−1^ of ^32^P-phosphate tracer (carrier free, Hartmann Analytic) was added simultaneously with 0 nmol l^−1^ (control), 80 and 800 nmol l^−1^ of unlabelled phosphate in separate experiments to chase the pulse of the ^33^P-phosphate tracer. Samples were incubated for further 2 h before being fixed with 1% (w/v) PFA to determine the uptake of ^33^P and ^32^P tracers in total samples and by flow-sorted bacterioplankton cells. The radioactivities of the two tracers were de-convoluted based on the differences in their energy spectra.

Bacterioplankton cells were flow sorted from SYBR Green I DNA-stained samples. Tracer uptake by *Prochlorococcus*, SAR11 and mean of total bacterioplankton cells were determined (Figs 1, 3). At least four proportional numbers of cells were sorted to determine the mean cellular content of tracers. Sorted cells were collected onto 0.2 μm pore size polycarbonate filters^2^,^7^.

Microbial uptake rates of bioavailable phosphate and Avogadro's constant were used to convert relative cellular rates of phosphate uptake into absolute rates in units of phosphate atoms taken by an average cell of the flow-sorted population (PO_4_^3−^ molecules per cell per hour). The size of cellular phosphate buffer (P_i_B) (PO_4_^3−^ molecules per cell) was estimated using measurements of ^33^P and ^32^P uptake into flow-sorted cells as follows:


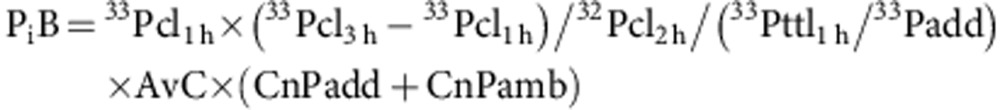


where ^33^Pcl_1 h_ is the cellular ^33^P uptake at the end of the 1-h pulse with 0.8 nmol phosphate per l (CnPadd), ^33^Pcl_3 h_ is the cellular ^33^P uptake at the end of the 1-h pulse with 0.8 nmol phosphate per l (CnPadd) added to the ambient bioavailable phosphate concentration (CnPamb) followed by 2-h chase with 80 nmol phosphate per l, ^32^Pcl_2 h_ is the cellular ^32^P uptake at the end of the 2-h chase with 80 nmol phosphate per l, ^33^Pttl_1 h_ is the total microbial ^33^P uptake per ml at the end of the 1-h pulse with 0.8 nmol phosphate per l and ^33^Padd is the total amount of added ^33^P tracer per ml.

### Modelling phosphate extracellular buffering

As a quick overview, the model tracks the uptake of radioisotope and stable phosphate tracers explicitly and separately. Phosphate is taken into the buffer from ambient water at a rate proportional to the concentration of phosphate in ambient water. The uptake rate is scaled by the degree to which the buffer is full. As the simplest option, this scaling is taken to decrease linearly with increasing phosphate in buffer until it and the uptake rate are zero at the designated maximum capacity. Once again for simplicity, it is assumed that the cell has a constant demand for phosphate and that there is a constant loss of phosphate from the cell. There is assumed to be no distinction between phosphate involving ^31^P, ^32^P and ^33^P in any process (Supplementary Figs 3a–b,7). Furthermore, it is assumed that the number of cells remains constant over the 3 h of the ‘experiment'.

The equations for the model are:


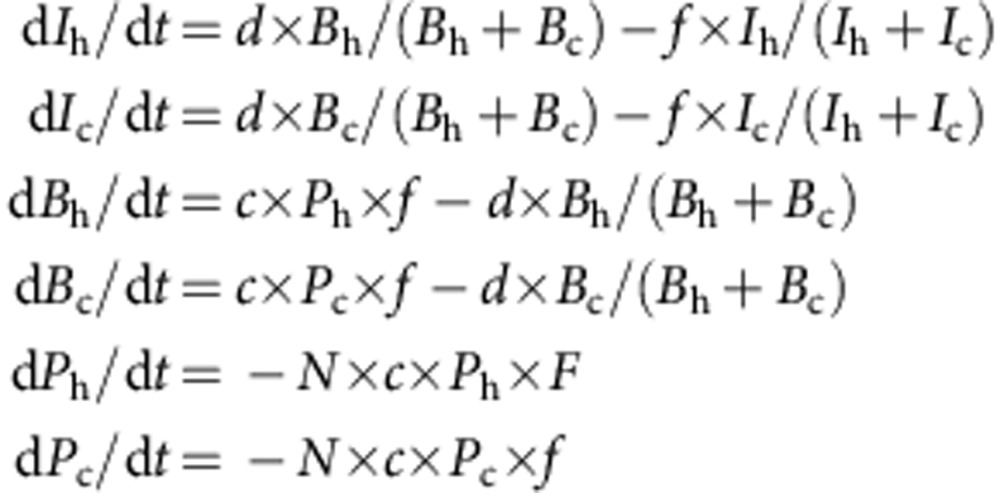


where *I* is phosphate internal to the cell, *B* is phosphate in the buffer, *P* is dissolved phosphate in the ambient water, h denotes ‘hot' phosphate (that is, involving ^32^P or ^33^P), c denotes ‘cold' phosphate (that is, involving ^31^P), *N* is the number of cells in 1 m^3^ and *F*=1−(*B*_h_+*B*_c_)/*B*_max_ is a function representing the extent to which the buffer is full. The units of the model are molecules of phosphate and hours, and the model represents 1 m^3^ of water. *B*_max_, *c*, *d* and *f* are constants taking the same values for all experiments at both sites. *B*_max_ represents the maximum number of molecules of phosphate that can be accommodated in the buffer of one cell, *c* (unit, per hour) is the maximum specific rate of uptake of phosphate from ambient waters into the buffer, *d* (unit, molecules per hour) controls the rate at which phosphate is taken from the buffer into the cell and *f* (unit, molecules per hour) is the rate at which phosphate is lost from the cell. The initial conditions for the model, including the number of cells, are taken from the experiments and the model is run for the same 3-hour period, with ‘hot' and ‘cold' tracers being added in quantities and at times once more matching the observations. There are seven parameters in the model. Their role is described in Supplementary Table 1. The model was solved using ode15s in Matlab.

The model is merely intended as a demonstration that the simple assumptions described above are qualitatively consistent with the observations; in particular, that the presence of a buffer allows the model to capture the relative changes in uptake rates seen in the observations. However, many of the parameters are poorly known, if at all. Therefore, to reduce the time involved in searching through parameter values one by one, we use the microgenetic algorithm^27^ for fitting the model to the data. The imposed parameter ranges are shown in Supplementary Table 1. In the absence of direct data the rates are chosen to cover a broad range. Data are available for ambient concentrations but once again a broad range encompassing the observations to ensure against uncertainties associated with measuring such low concentrations. If the optimizer had resulted in parameter values at the limits of the range, the ranges would have been expanded and the optimizer re-run. This did not prove necessary. Note that we do not use the optimization to claim the results presented are the best fit to the data. The optimization method is purely used to save time exploring whether there is a solution of the model, which resembles the observations. Without the presence of the buffer, the optimizer was unable to find such a solution despite several independent runs of the optimizer.

## Additional information

**How to cite this article:** Zubkov, M. V. *et al.* Dominant oceanic bacteria secure phosphate using a large extracellular buffer. *Nat. Commun.* 6:7878 doi: 10.1038/ncomms8878 (2015).

## Supplementary Material

Supplementary Information Supplementary Figures 1-8 and Supplementary Table 1
