# Supplementary material for: Dominant oceanic bacteria secure phosphate using a large extracellular buffer
Source: Nat Commun. 2015 Jul 22;6:7878. doi: 10.1038/ncomms8878 (PMC4525184; doi:10.1038/ncomms8878)
Supplement: Supplementary Information — Supplementary Figures 1-8 and Supplementary Table 1 [file ncomms8878-s1.pdf]

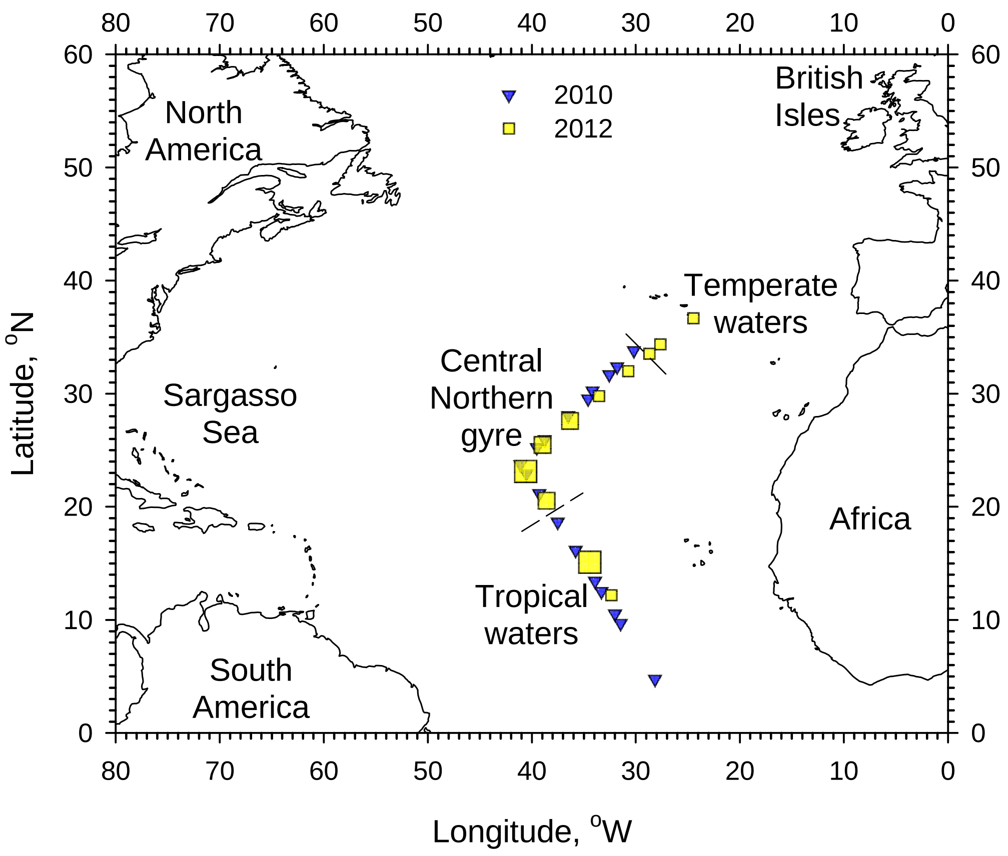

Supplementary Figure 1. Cruise tracks on AMT20 and AMT22

Cruise tracks of the Atlantic Meridional Transect (AMT) in 2010 & 2012 with station positions indicated as symbols. Small symbols indicate stations at which phosphate bioassays were carried out. Large and medium yellow squares indicate stations at which pulse-chase experiments were done. Additionally, large squares indicate stations at which phosphate acquisition data were modelled. Short dashed lines indicate the boundaries separating the central part of the North Atlantic subtropical gyre, characterised by fast (<10 h) microbial phosphate turnover (Supplementary Fig. 4c), from the temperate and tropical waters.

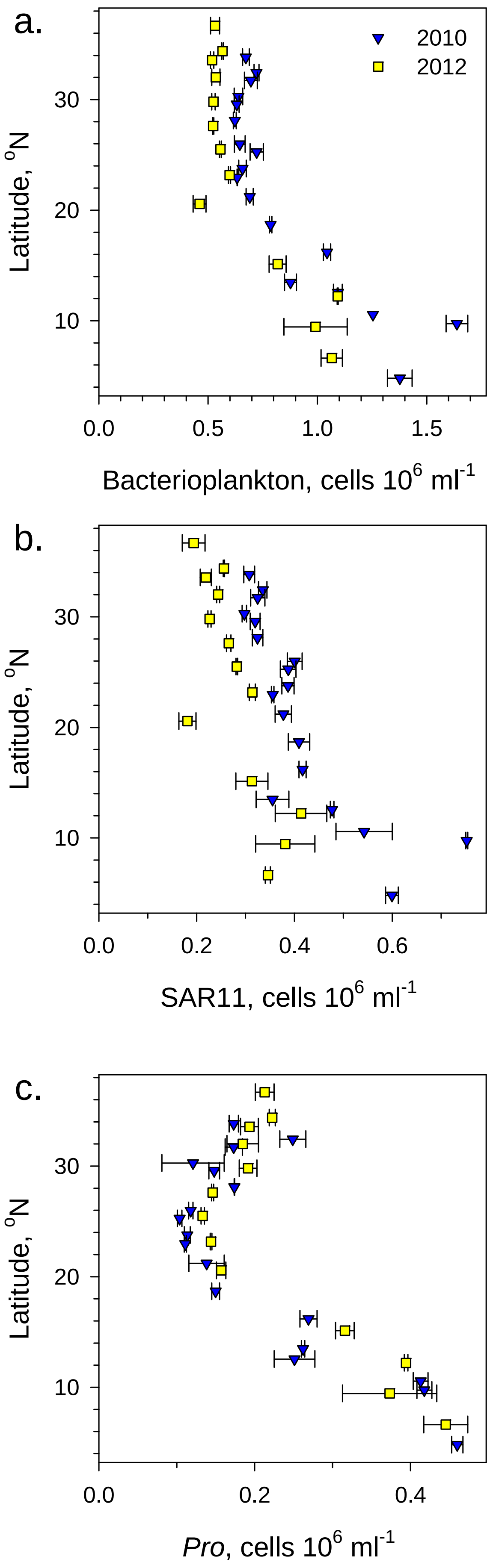
a.
2010
2012
Latitude, oN
30
20
10
0.0
0.5
1.0
1.5
Bacterioplankton, cells 10^6 ml^-1
b.
Latitude, oN
0.0
0.2
0.4
0.6
SAR11, cells 10^6 ml^-1
c.
Latitude, oN
0.0
0.2
0.4
Pro, cells 10^6 ml^-1

Supplementary Figure 2. Cellular abundance of dominant bacterioplankton groups along the latitudinal transects

Latitudinal distribution of cell abundance of total bacterioplankton (a), SAR11 Alphaproteobacteria (b) and *Prochlorococcus* cyanobacteria (c) on the 2010 and 2012 cruises. Changes in bacterioplankton abundance in surface waters (20 m sampling depth was chosen as a representative depth of the photic layer unaffected by the ship's presence) showed a similar latitudinal trend on the two cruises, with consistently lower cell abundance of total bacterioplankton, primarily owing to lower cell numbers of SAR11 although the cell numbers of the second most abundant bacterioplankton group *Prochlorococcus* were similar.

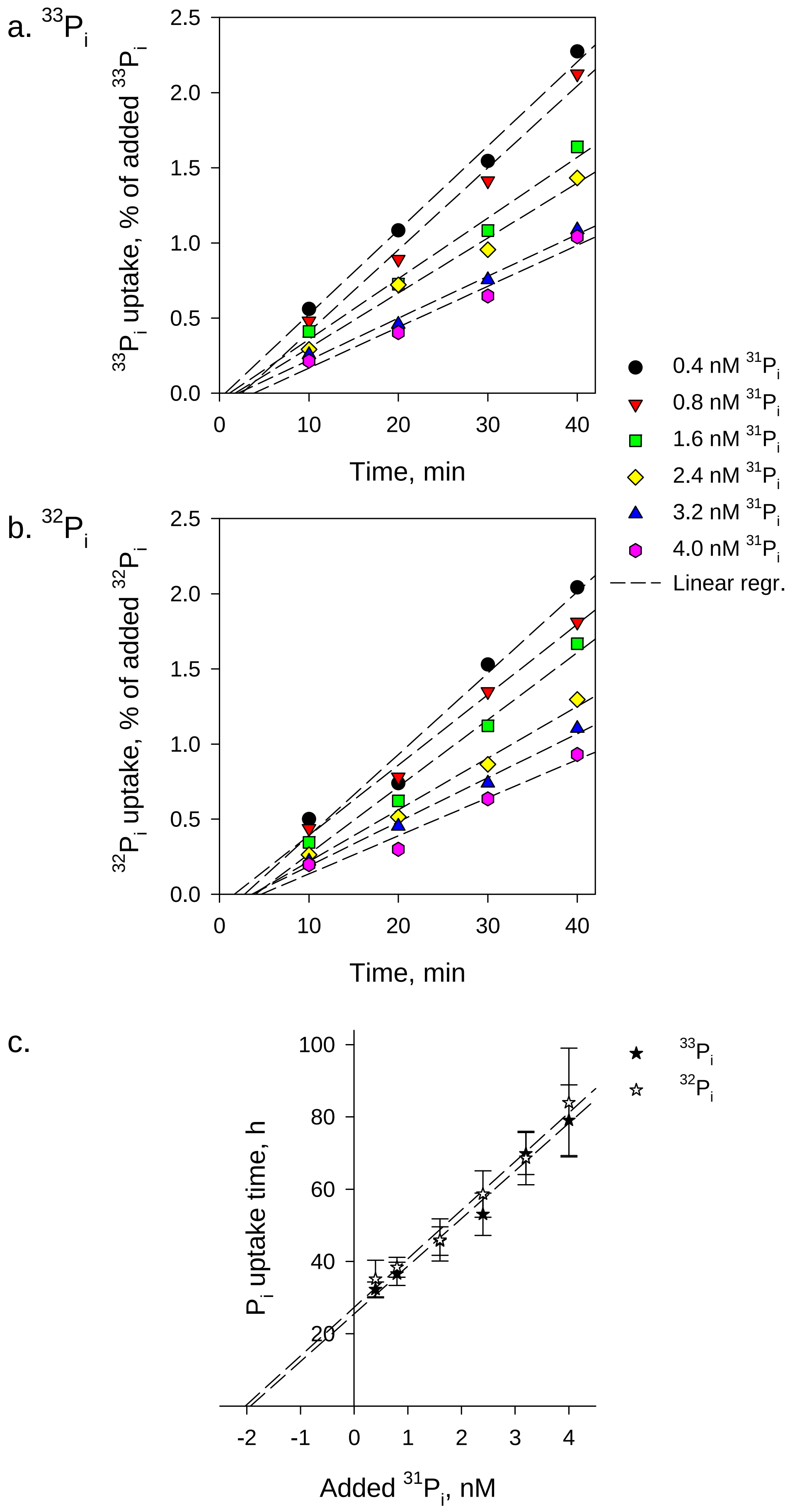

a. $^{33}P_i$
$^{33}P_i$ uptake, % of added $^{33}P_i$
0.0
0.5
1.0
1.5
2.0
2.5
0
10
20
30
40
Time, min
b. $^{32}P_i$
$^{32}P_i$ uptake, % of added $^{32}P_i$
0.0
0.5
1.0
1.5
2.0
2.5
0
10
20
30
40
Time, min
0.4 nM $^{31}P_i$
0.8 nM $^{31}P_i$
1.6 nM $^{31}P_i$
2.4 nM $^{31}P_i$
3.2 nM $^{31}P_i$
4.0 nM $^{31}P_i$
Linear regr.
c.
P_i uptake time, h
20
40
60
80
100
-2
-1
0
1
2
3
4
Added $^{31}P_i$, nM
$^{33}P_i$
$^{32}P_i$

Supplementary Figure 3. Comparison of bioassay estimation of ambient phosphate concentration, microbial uptake rate and uptake time using $^{33}P_i$ and $^{32}P_i$ tracers

a. Time series at different phosphate concentrations with corresponding regression lines (Linear regr.). Microbial phosphate uptake was estimated in a dilution series, in which $^{33}P_i$ at 0.05 nM was diluted with different amounts of non-labelled $^{31}P_i$ phosphate.

b. Time series at different phosphate concentrations with corresponding regression lines. Microbial phosphate uptake was estimated in a dilution series, in which $^{32}P_i$ at 0.01 nM was diluted with different amounts of non-labelled $^{31}P_i$ phosphate.

c. The relationships between added $^{31}P_i$ concentration and phosphate uptake time determined using $^{33}P_i$ (a) and $^{32}P_i$ (b) tracers and approximated using linear regressions with corresponding regression coefficient of 0.99 for both the $^{33}P_i$ and $^{32}P_i$ tracers. Error bars show propagated single standard errors. The ordinate intercepts of the linear regression lines in plot (c) are the estimates of microbial uptake time (25.5±2.5 h for $^{33}P_i$ and 27.3±2.6 h for $^{32}P_i$, respectively) at ambient phosphate concentrations, which are the abscissa (1.93±0.19 nM for $^{33}P_i$ and 2.03±0.19 nM for $^{32}P_i$, respectively). The estimates made using the two tracers are statistically similar.

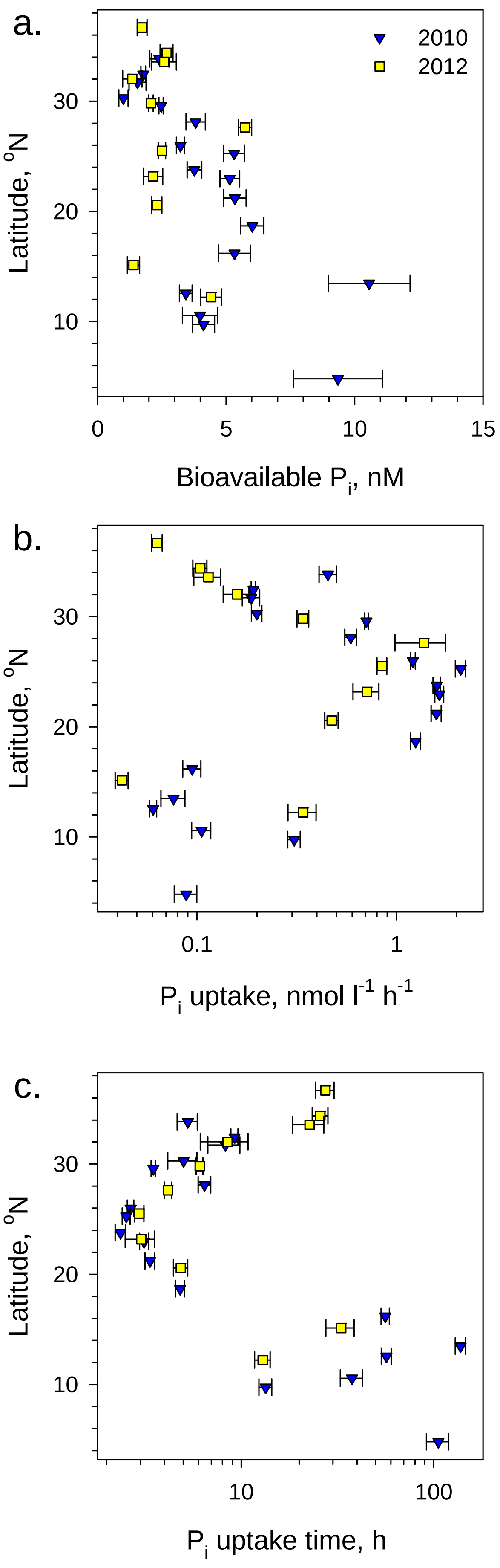
a.
2010
2012
Latitude, oN
Bioavailable Pi, nM
b.
Latitude, oN
Pi uptake, nmol l-1 h-1
c.
Latitude, oN
Pi uptake time, h

Supplementary Figure 4. Bioavailable concentrations, microbial uptake and turnover times of inorganic phosphate

Latitudinal changes in concentrations (a) of bioavailable inorganic phosphate (P_i) were also similar on the 2010 and 2012 cruises although concentrations were halved in the centre of the gyre on the later cruise. The latitudinal bell-shape changes in microbial uptake of phosphate (b) were generally similar with an order of magnitude lower uptake towards the gyre periphery. The parabolic latitudinal changes in the phosphate uptake (turnover) time (c) with vertex minimum in the gyre centre were very similar.

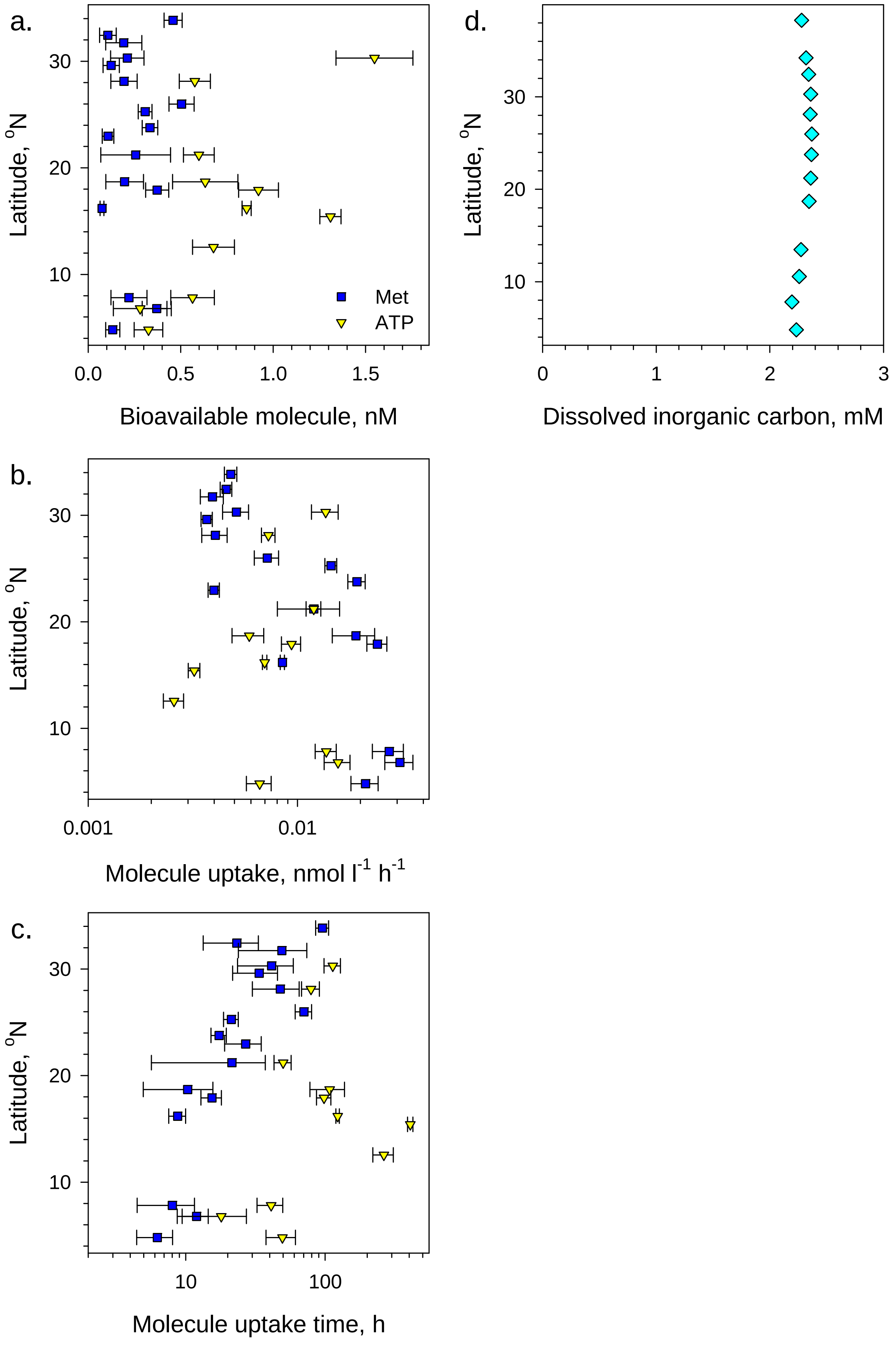
a.
Latitude, °N
30
20
10
Met
ATP
0.0
0.5
1.0
1.5
Bioavailable molecule, nM
d.
Latitude, °N
30
20
10
0
1
2
3
Dissolved inorganic carbon, mM
b.
Latitude, °N
30
20
10
0.001
0.01
Molecule uptake, nmol l-1 h-1
c.
Latitude, °N
30
20
10
10
100
Molecule uptake time, h

Supplementary Figure 5. Latitudinal changes in bioassay estimates of ambient methionine (Met), ATP and dissolved inorganic carbon (DIC) concentrations, microbial uptake rate and uptake time.

a. Comparison of bioavailable Met and ATP concentrations.

b. Comparison of microbial Met and ATP uptake rates.

c. Comparison of microbial Met and ATP uptake (turnover) time.

d. Concentrations of DIC.

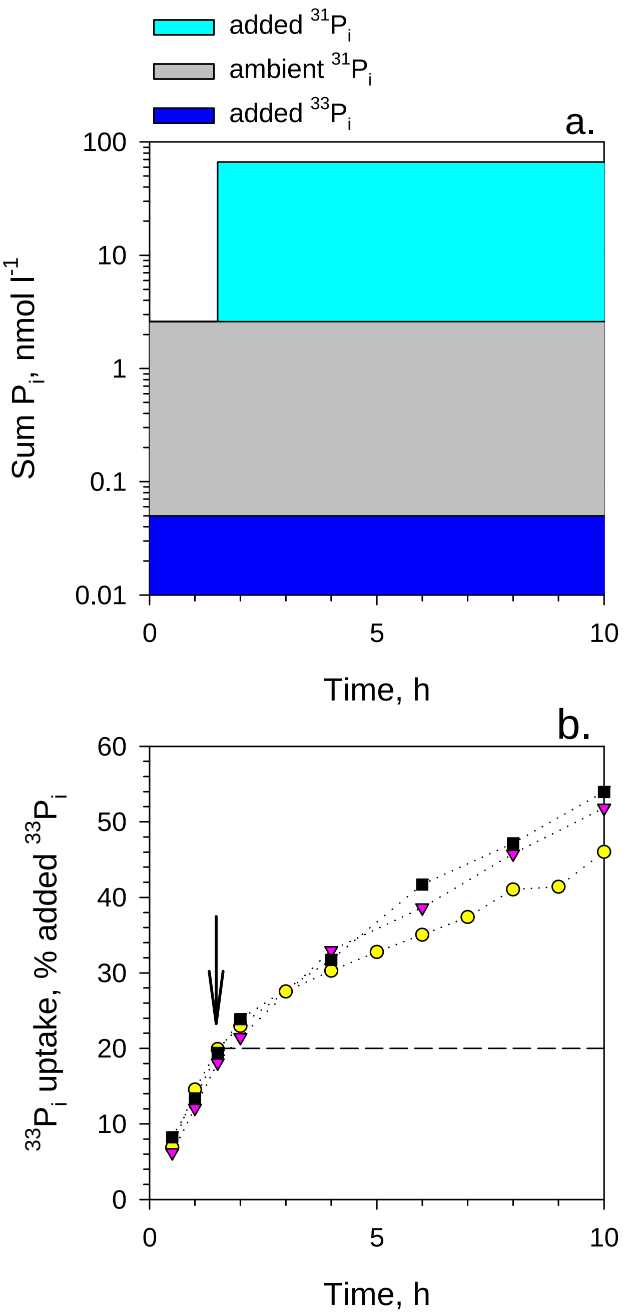

Supplementary Figure 6. Typical design of phosphate pulse-chase experiments

A pulse of 0.05 nmol l^{-1} $^{33}P_i$ added to ambient $^{31}P_i$ was chased by 64 nmol l^{-1} of unlabelled $^{31}P_i$ after 1.5 hours (a). Results of the three experiments to determine microbial uptake of pulse-chased $^{33}P_i$ (b) in the gyre centre. The chase addition is indicated by an arrow. The dashed line indicates the anticipated dynamics of $^{33}P_i$ after chase addition in the absence of the buffer.

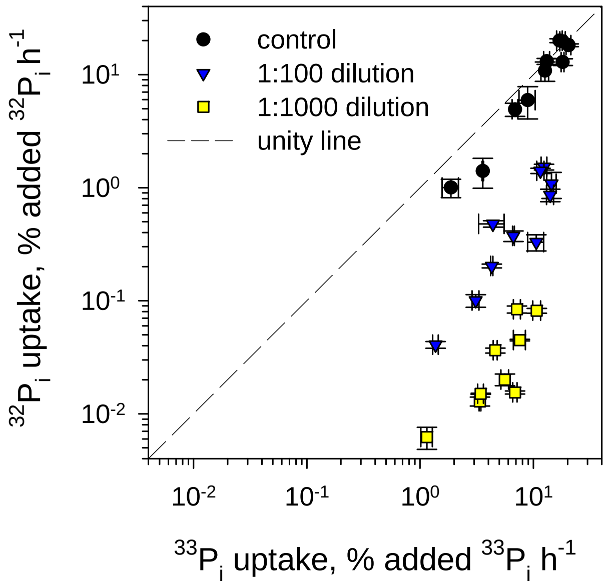

Supplementary Figure 7. $^{33}P_i$ and $^{32}P_i$ tracer uptake rates using different concentrations of $^{32}P_i$ in chase

Comparison of $^{33}P_i$ and $^{32}P_i$ tracer uptake in dual label pulse-chase experiments in undiluted control and chase dilutions labelled with $^{32}P_i$ tracer (Fig. 2).

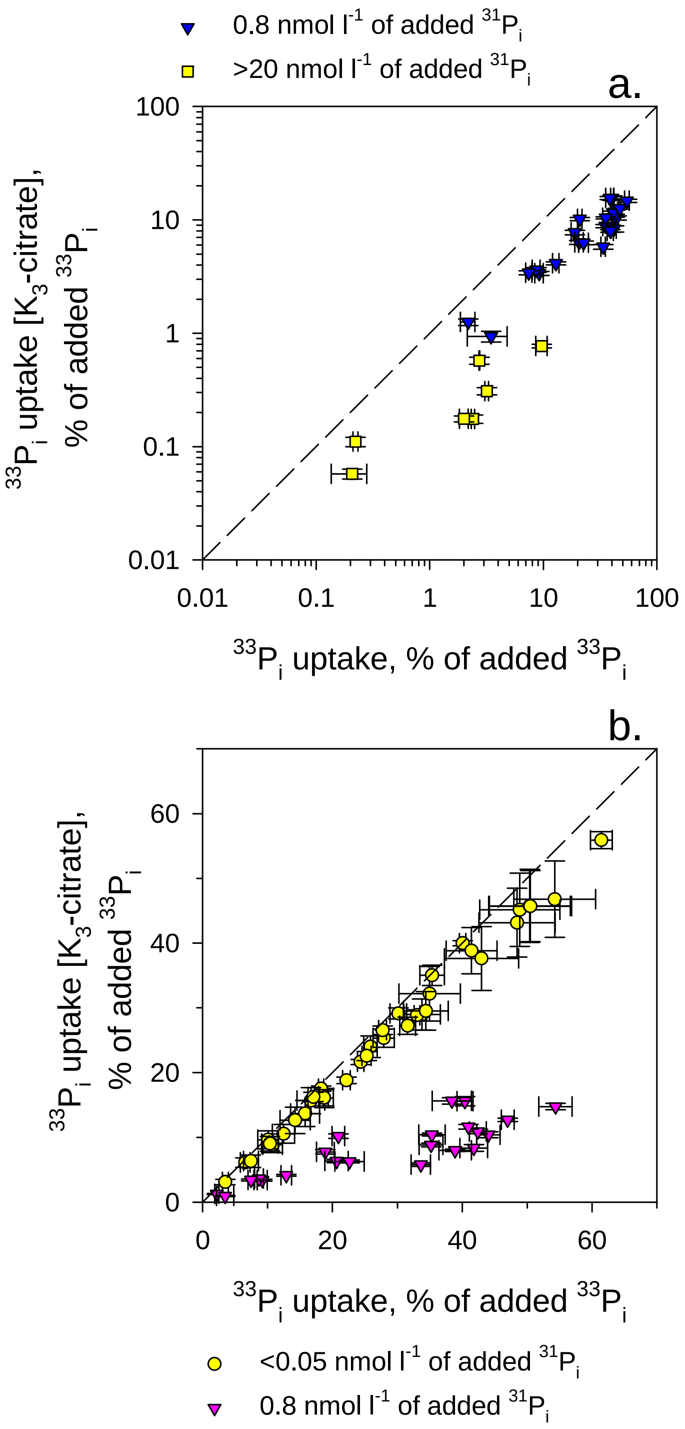

Supplementary Figure 8. Phosphate retention by fixed bacterioplankton

Comparison of phosphate retained by paraformaldehyde fixed bacterioplankton with and without tri-potassium-citrate (K_3-citrate) wash after labelling at different added phosphate concentrations.

	**Parameter**	**units**	**minimum**	**maximum**
**c**	Specific rate of PO_4 uptake into buffer	$hour^{-1}$	10^{-11}	10^{-9}
**d**	Rate of transfer of PO_4 from buffer to cell	molecules $hour^{-1}$	10^4	10^6
**f**	Rate of loss of PO_4 from cell	molecules $hour^{-1}$	10^3	10^5
$\mathbf{B_{max}}$	Size of buffer	molecules	10^6	10^8
**N**	Number of molecules of PO_4 in 1 m^3	N/A	10^{17}	$6x10^{18}$
	Initial fraction $(B_h+B_c)/B_{max}$	N/A	0	1

Supplementary Table 1. **Model parameters**

Description of parameters in the model, their units as well as the ranges used for the optimisation.
